# Ambient polycyclic aromatic hydrocarbon exposure and breast cancer risk in a population-based Canadian case–control study

**DOI:** 10.1007/s10552-024-01866-4

**Published:** 2024-04-17

**Authors:** Patrick Hinton, Paul J. Villeneuve, Elisabeth Galarneau, Kristian Larsen, Deyong Wen, Jun Meng, Verica Savic-Jovcic, Junhua Zhang, Will D. King

**Affiliations:** 1 Department of Public Health Sciences, Queen’s University, Kingston, ON Canada; 2 Department of Neuroscience, Carleton University, Ottawa, ON Canada; 3 Air Quality Research Division, Environment and Climate Change Canada, Toronto, ON Canada; 4 Office of Environmental Health, Health Canada, Ottawa, ON Canada

**Keywords:** Breast cancer, Polycyclic aromatic hydrocarbons, Air pollution, Case–control study

## Abstract

**Purpose:**

Polycyclic aromatic hydrocarbons (PAHs) represent a class of ubiquitous pollutants recognized as established human carcinogens and endocrine-disrupting chemicals. PAHs have seldom been modeled at the population-level in epidemiological studies. Fluoranthene is a prevalent PAH in urban settings and correlates with the occurrence of other PAHs. The purpose of this study was to evaluate associations between long-term residential exposure to ambient PAHs and breast cancer risk, both pre- and post-menopausal, in Canada.

**Methods:**

Using the National Enhanced Cancer Surveillance System (NECSS), a national-scale Canadian population-based case–control study, annual fluoranthene exposures were estimated using the GEM-MACH-PAH chemical transport model on the basis of geocoded residential histories throughout a 20-year exposure window. Odds ratios (ORs) and 95% confidence intervals (CIs) controlling for potential confounders were estimated using logistic regression. Separate analyses were conducted for Ontario and national samples given a finer-resolution exposure surface and additional risk factor information available for Ontario.

**Results:**

Positive associations were observed between fluoranthene exposure and premenopausal breast cancer, with inconsistent findings for postmenopausal breast cancer. For premenopausal breast cancer, adjusted ORs of 2.48 (95% CI: 1.29, 4.77) and 1.59 (95% CI: 1.11, 2.29) were observed when comparing the second highest category of exposure to the lowest, among the Ontario and national samples, respectively. For postmenopausal breast cancer, adjusted ORs were 1.10 (95% CI: 0.67, 1.80) and 1.33 (95% CI: 1.02, 1.73). Associations for the highest level of exposure, across both samples and menopausal strata, were non-significant.

**Conclusion:**

This study provides support for the hypothesis that ambient PAH exposures increase the risk of premenopausal breast cancer.

**Supplementary Information:**

The online version contains supplementary material available at 10.1007/s10552-024-01866-4.

## Introduction

The global incidence of female breast cancer is rising [1, 2], and in 2020, among women, breast cancer surpassed lung cancer in the number of incident cases reported worldwide [1, 3]. In Canada, breast cancer is the most commonly diagnosed cancer among women, and the second most commonly diagnosed cancer across both sexes [4, 5]. Despite the lengthy list of established and potential breast cancer risk factors [6–14], primarily behavioral, reproductive, or genetic in nature, these factors cannot fully explain many breast cancer cases, and some women who develop breast cancer will possess few of these known risk factors [15, 16]. Furthermore, the nature of the association between breast cancer risk factors and female breast cancer varies substantially by menopausal status and tumor subtype [10]. As a result, the etiology of breast cancer warrants further understanding, particularly with regard to long-term environmental exposures, which have garnered increasing attention due to suggestive animal and epidemiologic evidence [17, 18].

Ambient polycyclic aromatic hydrocarbons (PAHs), formed during the incomplete combustion of organic materials, represent a class of important ubiquitous pollutants recognized as animal and human carcinogens, mutagens, and teratogens [19–21]. In Canada, major anthropogenic sources of ambient PAH emissions include residential firewood combustion, vehicular/transportation-related emissions, and industrial plants, whereas virtually the entirety of natural Canadian PAH emissions are produced from forest fires [22]. While anthropogenic sources of ambient PAHs dominate human exposure in urban areas, the increasing frequency and severity of Canadian forest fires, in part due to the effects of climate change, may increasingly contribute to urban exposures, especially given the established long-range atmospheric transport capabilities of ambient PAHs [23, 24].

In terms of biological mechanisms of action, inhaled PAHs are able to enter the bloodstream through interstitial spaces between alveoli and subsequently associate with adipose-dense tissues due to their highly lipophilic properties [25]. Once in these tissues, reactive metabolites have the potential to generate reactive oxygen species in the cellular environment and DNA damage through the formation of DNA adducts [26, 27]. Additionally, PAHs are established endocrine-disrupting chemicals and have, more recently, been implicated as xenoestrogens (i.e., substances that mimic estrogens) [28, 29]. Rodent and mechanistic studies have supported the assumption that PAHs can contribute to the formation of mammary tumors [30].

Epidemiological research examining air pollutant exposures and cancer incidence has historically focused on commonly-monitored criteria air pollutants (e.g., fine particulate matter, PM_2.5_; nitrogen dioxide, NO_2_) and respiratory cancers [31–33]. More recently, characterizing the relationships between common non-respiratory cancer sites (breast, prostate, colorectal, etc.) and air pollutants, including less-frequently studied constituents, has garnered increasing interest as support for their associations appears to grow [34]. A recently published meta-analysis examining the relationship between NO_2_, a ubiquitous pollutant closely tied to PAHs and a marker for traffic-related air pollution, and breast cancer risk yielded a significant association (pooled relative risk (RR) per 10 µg/m^3^ = 1.015; 95% CI: 1.003, 1.028) [35].

In 2022, a systematic review and meta-analysis of the existing non-ecological research examining the relationship between [ambient and non-ambient] PAH exposures and breast cancer risk was published [36]. A summary relative risk estimate from five studies that specifically examined outdoor ambient PAH exposures, all assessing vehicular and traffic-related exposures, was not statistically significant [36].

Given the limited existing findings between breast cancer risk and outdoor ambient PAH exposure, and suggestive evidence for criteria pollutant exposures, further research that incorporates improved and sophisticated PAH exposure characterization methods is needed. Likewise, additional research continues to be required to distinguish how the relationship between air pollutants and breast cancer risk differs according to menopausal status given inconsistent findings [37–39]. The current study evaluates associations between long-term residential exposure to ambient PAHs and breast cancer risk in the Canadian setting. We also examine how the relationship between ambient PAH exposures and breast cancer risk differs according to menopausal status. This study adds to the current literature by assessing modeled long-term PAH exposure to both pre and postmenopausal breast cancer risk in Canada.

## Methods

### Case–control study design

The current study draws its study population from the Canadian National Enhanced Cancer Surveillance System (NECSS), a collaborative effort between Health Canada and the Canadian Provincial Cancer Registries, with data collection starting in 1994 [40]. The NECSS, conducted in eight of the 10 Canadian provinces (all except Quebec and New Brunswick), contains rich and comprehensive individual-level risk factor data for a large population-based Canadian case–control study of 18 different cancer sites and includes ~ 5,000 population controls [40, 41]. Importantly, the NECSS collected individual-level data regarding lifetime residential histories. All participants within the NECSS provided informed consent prior to being included. Due to additional covariate information collected by the province of Ontario, along with the existence of a finer-resolution component of the PAH exposure surface available, the current study reports main analyses based on both the national (i.e., all eight participating provinces) and Ontario-only samples.

Incident breast cancer cases were identified starting in 1994 through the respective provincial cancer registries by randomly sampling one in four eligible participants with newly diagnosed histologically-confirmed invasive primary breast cancer, as defined by the *International Classification of Diseases* [42]. Provincial registries identified patients within one to four months from their diagnosis through the National Cancer Incidence Reporting System. The provincial registries ensured physician consent was given before approaching breast cancer cases. Sampling was performed for each year until a population-based quota was met, resulting in a total study period spanning 1994–1997. All cases were women aged 25–74 at the time of cancer diagnosis. Premenopausal women with breast cancer were over-sampled to ensure adequate power when exploring relationships with risk factors across menopausal strata [40]. Initially, 3,310 female breast cancer cases were ascertained across the participating provinces. Due to a host of factors, namely physician refusals and case deaths, a total of 3,023 questionnaires were mailed to cases, and of these, 2,340 were successfully completed and returned, yielding a case participation rate of ~ 77.4%.

NECSS population controls were identified in 1996 by each participating provincial cancer registry via frequency-matching on the basis of age and sex for the overall distribution of cases, across all 18 cancer sites (i.e., types of primary cancer) included within NECSS [40]. The specific random sampling methods used to obtain population controls differed across participating provinces according to accessibility and availability of data, details can be found elsewhere [40, 41, 43, 44]. Ascertainment of information from the controls was performed per the same protocol as for the NECSS cancer cases.

Questionnaires were successfully mailed out to 3,550 potential controls and 2,531 completed questionnaires were returned (71.3%). Both case and control participation rates were similar in the Ontario sample.

### Data collection

Information regarding participant risk factors and residential histories were collected via mailed questionnaires. Specific information collected through these questionnaires included a broad set of demographic, lifestyle, and environmental factors. Namely, information was ascertained regarding; family income, education, marital status, employment history, residential history, reproductive-related factors, body mass index (BMI), smoking history, alcohol consumption, dietary history, physical activity, and vitamin and mineral supplements (among others) [40, 41]. The dietary history component of the questionnaires was based on previously validated instruments [43, 45, 46]. The questionnaire also included specific questions regarding established and potential risk factors for breast cancer.

The NECSS questionnaire delivered in Ontario included additional information for a number of breast cancer risk factors (e.g., oral contraceptive use, hormone replacement therapy, family history of cancer, benign breast disease) not collected in the other provinces. Additionally, a few factors (namely physical activity) were assessed and measured via different methods when comparing the Ontario NECSS to the other versions of the NECSS, thereby requiring harmonization of these measures for national analyses. For additional information regarding the NECSS design and data collection procedures, refer to Johnson et al. [40].

The sole potential adjustment factor that we sourced via outside (non-NECSS) means was a quintile index of neighborhood deprivation (for the year 1996) [47, 48]. This measure was linked to participant longest residence (at any time) and was sought-out based on the considerations that; (1) individual-level measures of socio-economic status (SES), such as income and education, may not be able to fully capture all aspects of SES (resulting in residual confounding), and (2) levels of air pollution are typically greater in areas of lower-SES [49], and women residing in areas of high-SES may be more at risk for developing breast cancer (i.e., qualification as a potential confounder) [50].

Residential histories included complete addresses and corresponding six-character Canadian postal codes. The postal code centroid was used to represent home for all respondents. All valid six-character postal codes were geocoded to the geographic center of postal codes as of 1996 [51]. The home postal code subsequently represents the spatial basis for residential PAH exposure assignment. Of note, six-character postal codes in densely-populated urban areas often represent quite small domains, whereas in rural areas the domain covered by postal codes may be much larger. Based on postal code classifications (i.e., second digit 0 means rural), rural areas within the eight sampled provinces had a median size of 0.018 km^2^ (mean = 7.9 km^2^), while urban areas had a median size of 0.008 km^2^ (mean = 0.14 km^2^). On average, PAH concentrations are more homogeneous and orders of magnitude lower in rural versus urban areas. As a result, the measurement error introduced by the larger size of rural postal codes is unlikely to introduce substantial misclassification of PAH exposure.

### Assessment of exposure to ambient concentrations of PAHs

The exposure time period was a 20-year window back from 2 years prior to diagnosis (cases) or recruitment (controls) [52]. The GEM-MACH-PAH (Global Environmental Multiscale model – Modeling Air quality and Chemistry—Polycyclic Aromatic Hydrocarbons) model generated PAH surfaces using emissions data from the year 2000 (earliest year available) paired with meteorologic data from the year 1994. Meteorologic conditions in 1994 were found to best represent the average meteorology of the preceding decade (1990–2000). Subject residence history was linked to PAH surfaces using the centroid of the 6-digit postal code on a year-by-year basis. Inclusion criteria required that participants provided at least 16-years’ (i.e., 80% of the exposure window) of residential histories and exposure was averaged over this window for available years of residence. Inclusion criteria restrictions reduced the sample size to 1,233 (514 cases; 719 controls) for Ontario analyses and 3773 (1818 cases; 1955 controls) for national analyses.

### GEM-MACH-PAH model

**Fig. 1:**
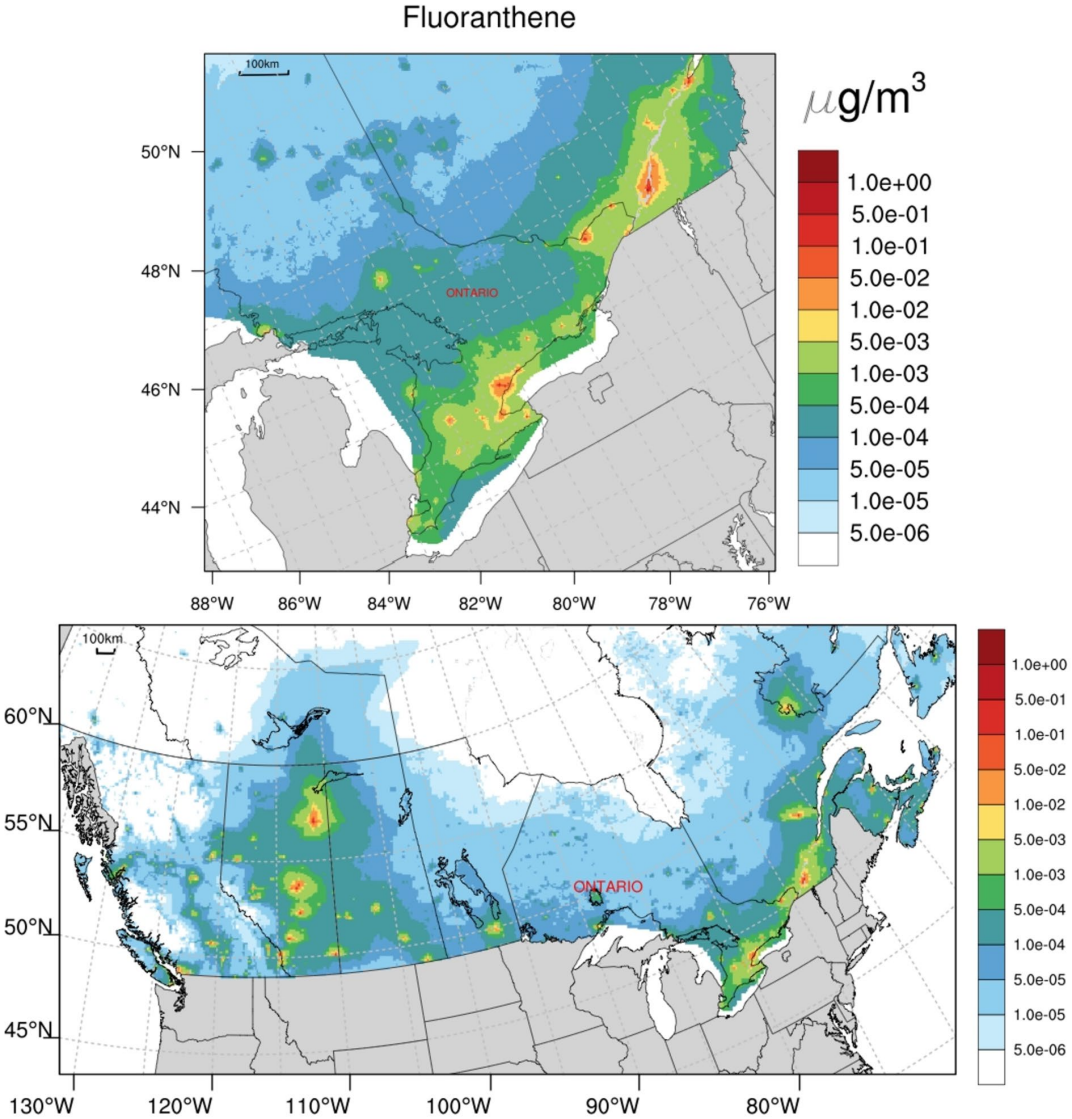
Ambient fluoranthene concentration estimates and spatial distribution (Year 2000) generated from GEM-MACH-PAH chemical transport model, Canadian domain (bottom; 10 km × 10 km model resolution) and nested “Pan Am” domain (top; 2.5 km × 2.5 km model resolution)

The PAH exposure surface (Fig. 1) was generated by the GEM-MACH-PAH chemical transport model [53, 54]. The previously validated GEM-MACH-PAH model simulates airborne PAH concentrations from estimated emissions that are transported and transformed by modeled meteorology and atmospheric processes (e.g., oxidation, deposition, etc.). GEM-MACH-PAH was initially run for a 10 km × 10 km horizontal grid square domain over continental Canada/United States that subsequently drove the boundary conditions for a smaller 2.5 km × 2.5 km domain (“Pan Am” domain) centered on the eastern Laurentian Great Lakes. This finer resolution domain formed the basis for Ontario-specific main analyses. Gridded pollutant emissions were generated for the year 2000 (the earliest year possible), and these were paired with meteorology from the year 1994 which was found to best represent average temperatures and precipitation for the two preceding decades. Fluoranthene was used as a representative PAH due to its high modeled accuracy compared to measurements, its prevalence in urban PAH air pollution, and its presence in both the gas and particle phases of ambient air [53]. Output from validated simulations demonstrates a high degree of spatial correlation among individual PAH compounds (Online Resource 1; Table S2) thereby allowing a single compound to represent PAHs as a class [53, 54].

### Statistical analyses

All analyses, except for the generation of spline curves, were conducted using SAS, version 9.4 (SAS Institute, Inc., North Carolina). Restricted cubic spline curves were created using R Statistical Software (v4.3.0; R Core Team 2023).

We used unconditional logistic regression to estimate the odds ratios (ORs) and 95% confidence intervals (CIs) for breast cancer incidence associated with mean fluoranthene exposure levels across the study exposure period. We conducted main analyses using three principal stratifications; all women grouped together, only women of premenopausal status, and only women of post-menopausal status. For the current study, premenopausal status was assigned to [female] participants if, at the time of completing the questionnaire, they were; (1) still menstruating, or (2) less than 50 years old and had an unreported menstruation status, or (3) not currently menstruating but last menstruation reported within the preceding year, otherwise, postmenopausal status was assigned.

Exposure–response patterns for continuous covariates were explored using Box-Tidwell tests and qualitative examination of age-adjusted effect estimates based on equidistantly-spaced categorical representations [55]. Where non-linear relationships of the logit were present, or if linearity was called into question, categorical representations were considered for main analyses. Specifically, categorical cut-points were determined based on the following prioritized criteria; (1) if previously established and common cut-points exist, these were used (e.g., BMI), (2) if the same covariate [in categorical form] were presented in prior studies utilizing the breast cancer component of the NECSS, these were used, (3) otherwise, quartile cut-points were used. For the main exposure of interest (fluoranthene), age-adjusted restricted cubic splines confirmed non-linearity of the logit (for all three stratifications), and thus a categorical representation was warranted. Ensuing, we determined cut-points for fluoranthene by creating equidistant levels of exposure based on the log-transformed variable (due to a highly right-skewed distribution) while ensuring that each level of exposure comprised at least 10% of the control distribution across both the Ontario- and national-analyses and when considering stratifications (i.e., pre- and post-menopausal). Appropriately, all crude and fully-adjusted ORs for breast cancer incidence were reported with respect to the lowest exposure level as the referent category.

Covariates considered for model adjustment were those with established or plausible relationships with breast cancer risk and meeting criteria for appropriateness as a confounder [14]. Since the relationships between risk factors for premenopausal and postmenopausal breast cancer were assumed a priori to be different [10], main analyses employed backwards elimination, with a p-value criterion of 0.2, to determine individual covariate sets for all three stratifications (all women, premenopausal only, postmenopausal only). This was done separately for both Ontario and national samples given the considerable difference in sample size and sample populations.

Due to the presence of missing data for multiple covariates (Table 1 and Online Resource 1; Table S1), and assuming the mechanism behind missingness was Missing at Random (MAR), we conducted single stochastic regression imputation [56, 57]. All analyses are presented with respect to the imputed covariates herein.

**Table 1:** Distribution of breast cancer risk factors by case–control and menopausal status, Ontario sample, (*n* cases = 514,* n* controls = 719)

Variable^a^	Pre-menopausal^b^		*p*c	Post-menopausal^b^		*p*c
	Cases [*n* (%)]	Controls [*n* (%)]		Cases [*n* (%)]	Controls [*n* (%)]	
Age group, years			0.04			0.16
20–29	3 (1.5)	14 (5.7)		0 (0)	0 (0)	
30–39	35 (16.9)	40 (16.4)		4 (1.3)	1 (0.2)	
40–49	123 (59.4)	124 (50.8)		26 (8.5)	33 (7.0)	
50–59 (ref.)	41 (19.8)	50 (20.5)		76 (24.8)	91 (19.2)	
60–69	5 (2.4)	16 (6.6)		121 (39.4)	215 (45.3)	
> 70	0 (0)	0 (0)		79 (25.7)	135 (28.4)	
Not Reported	0 (0)	0 (0)		1 (0.3)	0 (0)	
Body mass index (kg/m^2^)			0.23			0.02
< 18.5	9 (4.4)	6 (2.5)		7 (2.3)	6 (1.3)	
18.5 to < 25 (ref.)	136 (65.7)	145 (59.4)		146 (47.6)	249 (52.4)	
25 to < 30	40 (19.3)	69 (28.3)		84 (27.4)	153 (32.2)	
≥ 30	21 (10.1)	24 (9.8)		69 (22.5)	65 (13.7)	
Not Reported	1 (0.5)	0 (0)		1 (0.3)	2 (0.4)	
Years of education			0.27			0.08
< 11 (ref.)	24 (11.6)	39 (16.0)		97 (31.6)	173 (36.4)	
11–12	64 (30.9)	59 (24.2)		74 (24.1)	126 (26.5)	
13–14	47 (22.7)	47 (19.3)		56 (18.2)	80 (16.8)	
> 14	71 (34.3)	97 (39.8)		77 (25.1)	85 (17.9)	
Not Reported	1 (0.5)	2 (0.8)		3 (1.0)	11 (2.3)	
Total household income (CAD$)			0.48			0.07
< $50,000 (ref.)	59 (28.5)	74 (30.3)		135 (44.0)	218 (45.9)	
$50,000–$99,999	78 (37.7)	84 (34.4)		63 (20.5)	68 (14.3)	
≥ $100,000	27 (13.0)	24 (9.8)		8 (2.6)	24 (5.1)	
Not Reported	43 (20.8)	62 (25.4)		101 (32.9)	165 (34.7)	
Physical activity (standardized MET score quartiles)^d^			0.93			0.01
Q1 (< − 0.73) (ref.)	49 (23.7)	56 (30.0)		84 (27.4)	141 (29.7)	
Q2 (− 0.73 to − 0.12)	54 (26.1)	63 (25.8)		90 (29.3)	89 (18.7)	
Q3 (− 0.13 to 0.54)	45 (21.7)	47 (19.3)		81 (26.4)	130 (27.4)	
Q4 (> 0.54)	43 (20.8)	56 (30.0)		44 (14.3)	96 (20.2)	
Not Reported	16 (7.7)	22 (9.0)		8 (2.6)	19 (4.0)	
Index of neighborhood deprivation (quintiles)^e^			0.45			0.72
Q1 (Most deprived) (ref.)	49 (23.7)	45 (18.4)		61 (19.9)	80 (16.8)	
Q2	38 (18.4)	46 (18.9)		54 (17.6)	93 (19.6)	
Q3	45 (21.7)	58 (23.8)		74 (24.1)	106 (22.3)	
Q4	48 (23.2)	51 (20.9)		68 (22.2)	109 (23.0)	
Q5 (Least deprived)	27 (13.0)	44 (18.0)		50 (16.3)	87 (18.3)	
Smoking pack-years			0.20			0.20
0 (ref.)	97 (46.9)	132 (54.1)		149 (48.5)	257 (54.1)	
1	25 (12.1)	19 (7.8)		16 (5.2)	17 (3.6)	
2–13	52 (25.1)	48 (19.7)		45 (14.7)	73 (15.4)	
> 13	32 (15.5)	41 (16.8)		95 (30.9)	120 (25.3)	
Not Reported	1 (0.5)	4 (1.6)		2 (0.7)	8 (1.7)	
Alcohol consumption (units/week)			0.27			0.43
0 (ref.)	65 (31.4)	98 (40.2)		134 (43.7)	223 (47.0)	
< 0.5	23 (11.1)	26 (10.7)		35 (11.4)	50 (10.5)	
0.5–3.5	58 (28.0)	56 (23.0)		70 (22.8)	87 (18.3)	
> 3.5	61 (29.5)	64 (26.2)		68 (22.2)	115 (24.2)	
Meat consumption (servings/week; quartiles)			0.88			0.43
Q1 (< 3.87) (ref.)	48 (23.2)	61 (25.0)		77 (25.1)	138 (29.1)	
Q2 (3.87–6.40)	48 (23.2)	61 (25.0)		79 (25.7)	119 (25.1)	
Q3 (6.41–9.81)	57 (27.5)	65 (26.6)		75 (24.4)	121 (25.5)	
Q4 (> 9.81)	54 (26.1)	57 (23.4)		76 (24.8)	97 (20.4)	
Vegetable consumption (servings/week; quartiles)			0.48			0.94
Q1 (< 13.90) (ref.)	69 (33.3)	79 (32.4)		79 (25.7)	123 (25.9)	
Q2 (13.90–18.96)	60 (29.0)	65 (26.6)		75 (24.4)	110 (23.2)	
Q3 (18.97–25.46)	37 (17.9)	58 (23.8)		67 (21.8)	100 (21.1)	
Q4 (> 25.46)	41 (19.8)	42 (17.2)		86 (28.0)	142 (29.9)	
Parity			0.38			0.18
0 (ref.)	29 (14.0)	47 (19.3)		45 (14.7)	43 (9.1)	
1	24 (11.6)	22 (9.0)		23 (7.5)	40 (8.4)	
2	67 (32.4)	87 (35.7)		56 (18.2)	101 (21.3)	
3	49 (23.7)	46 (18.9)		78 (25.4)	109 (23.0)	
≥ 4	38 (18.4)	42 (17.2)		104 (33.9)	181 (38.1)	
Not Reported	0 (0)	0 (0)		1 (0.3)	1 (0.2)	
Ever breast fed			0.87			0.71
No (ref.)	63 (30.4)	76 (31.2)		100 (32.6)	168 (35.4)	
Yes	144 (69.6)	168 (68.9)		207 (67.4)	306 (64.4)	
Not Reported	0 (0)	0 (0)		0 (0)	1 (0.2)	
Age at first full-term pregnancy, years			0.13			< 0.01
< 18	8 (3.9)	5 (2.1)		11 (3.6)	12 (2.5)	
18–26 (ref.)	86 (41.6)	112 (45.9)		144 (46.9)	295 (62.1)	
27–30	63 (30.4)	56 (30.0)		69 (22.5)	94 (19.8)	
> 30	21 (10.1)	21 (8.6)		35 (11.4)	28 (5.9)	
Never Pregnant	29 (14.0)	50 (20.5)		48 (15.6)	46 (9.7)	
Years of menstruation (quartiles)			0.25			0.01
Q1 (< 28) (ref.)	41 (19.8)	61 (25.0)		45 (14.7)	104 (21.9)	
Q2 (28–33)	61 (29.5)	67 (27.5)		79 (25.7)	119 (25.1)	
Q3 (34–37)	56 (27.1)	47 (19.3)		66 (21.5)	85 (17.9)	
Q4 (> 37)	32 (15.5)	44 (18.0)		103 (33.6)	128 (27.0)	
Not Reported	17 (8.2)	25 (10.3)		14 (4.6)	39 (8.2)	
Age at menarche, years			0.18			0.11
< 12 (ref.)	50 (24.2)	36 (14.8)		50 (16.3)	81 (17.1)	
12	47 (22.7)	69 (28.3)		77 (25.1)	104 (21.9)	
13	52 (25.1)	65 (26.6)		84 (27.4)	101 (21.3)	
14	32 (15.5)	35 (14.3)		50 (16.3)	86 (18.1)	
> 14	15 (7.3)	24 (9.8)		34 (11.1)	70 (14.7)	
Not Reported	11 (5.3)	15 (6.2)		12 (3.9)	33 (7.0)	
Benign breast disease			< 0.01			< 0.01
No (ref.)	139 (67.2)	220 (90.2)		191 (62.2)	412 (86.7)	
Yes	25 (12.1)	5 (2.1)		42 (13.7)	31 (6.5)	
Not Reported	43 (20.8)	19 (7.8)		74 (24.1)	32 (6.7)	
Oral contraceptive use (≥ 6 months)			0.02			0.68
No (ref.)	46 (22.2)	80 (32.8)		202 (65.8)	316 (66.5)	
Yes	160 (77.3)	160 (65.6)		103 (33.6)	153 (32.2)	
Not Reported	1 (0.5)	4 (1.6)		2 (0.7)	6 (1.3)	
Immediate relative with cancer			0.01			0.11
No (ref.)	99 (47.8)	152 (62.3)		129 (42.0)	235 (49.5)	
Yes	106 (51.2)	91 (37.3)		174 (56.7)	236 (49.7)	
Not Reported	2 (1.0)	1 (0.4)		4 (1.3)	4 (0.8)	
Hormone Replacement Therapy (≥ 6 months)			0.13			0.26
No (ref.)	188 (90.8)	207 (84.8)		195 (63.7)	312 (65.7)	
Yes	18 (8.7)	32 (13.1)		111 (36.3)	156 (32.8)	
Not Reported	1 (0.5)	5 (2.1)		1 (0.3)	7 (1.5)	

*NE* not estimated, *MET* Metabolic Equivalent of Task

^a^Categories shown are those modeled in regression analyses, excluding ‘not reported’ categories

^b^Premenopausal women defined as women who were (at time of interview); (i) still menstruating, or (ii) menstruating status not reported and age less than 50, or (iii) not currently menstruating and last menstruation within previous year

^c^Bivariate p-value for breast cancer (Wald Chi-square test)

^d^Physical activity measure for Ontario standardized in harmonization with national analysis ^e^Quintiles based on single component (deprivation) of long-term neighbourhood socioeconomic status index associated with participant longest residence

### Nitrogen dioxide

In addition to main analyses, we performed a number of exploratory analyses involving NO_2_. Namely, we were interested in; (1) investigating the degree to which concentrations of NO_2_ are spatially correlated (for the year 2000) with GEM-MACH-PAH generated estimates of fluoranthene, and (2) to what extent do the individual associations between NO_2_ and fluoranthene with breast cancer risk, respectively, differ from those observed when these ambient pollutants are modeled together (i.e., controlling for each other).

To facilitate the aforementioned analyses, we sourced national pollutant estimates, for the year 2000, from Canadian Consortium on Urban Environmental Health (CANUE: www.canue.ca) data repositories [58]. Briefly, estimates for NO_2_ were generated from a national land-use regression (LUR) model using national air pollution surveillance (NAPS) monitoring data [59–61]. Estimates for NO_2_ (µg/m^3^) were linked to corresponding annual postal code files by CANUE. National ambient fluoranthene estimates (10 km model resolution) linked to postal codes used to derive average exposures for NECSS participants across residential histories (~ 8,100 individual postal codes) were used in conjunction with corresponding NO_2_ estimates (i.e., at corresponding postal codes) to drive correlation analyses. Spearman correlation was estimated at the level of individual postal codes, across Canada, for which we were able to obtain estimates for both fluoranthene and NO_2_ (7,300 postal codes).

To explore associations between NO_2_ and breast cancer risk (including combined NO_2_ and fluoranthene models), we created quintile-based exposure categories based on the control distribution (i.e., 20% of controls in each level of exposure) for the Ontario sample and ran logistic models with adjustment for the same covariates controlled for within the main Ontario analysis. This analysis was restricted to participants with valid average exposure measures for both NO_2_ and fluoranthene, reducing the sample to 494 cases and 681 controls.

## Results

The original breast cancer component of the NECSS dataset contained 2,340 cases and 2,531 controls. For Ontario (2.5 km × 2.5 km exposure grid), 514 cases and 719 controls met inclusion criteria based on completion of an Ontario version of the NECSS questionnaire, and completeness of residential history and menopausal status. For the national sample (10 km × 10 km exposure grid), a total 1,818 cases and 1,955 controls met inclusion criteria based on completeness of residential history and menopausal status.

### Description of cases and controls

Table 1 displays the covariate (risk factor) distributions and characteristics for all cases and controls within the Ontario sample. Table S1 (Online Resource 1) presents the same information with respect to the national sample.

Among the Ontario sample, the mean age of pre- and post-menopausal cases was 45.3 and 62.3, and 45.3 and 63.5 for controls, respectively (not shown in Table 1). In premenopausal women, cases tended to have somewhat higher household income, higher number of smoking pack-years, slightly less physical activity, higher alcohol consumption, higher proportion of benign breast disease, oral contraceptive use, and immediate relatives diagnosed with cancer, when compared to controls. In postmenopausal women, cases had higher BMI, more years of education, slightly less physical activity and number of children, older age at first full-term pregnancy (or never pregnant), higher number of years menstruated, younger age at menarche, and a higher proportion of benign breast disease and immediate relatives diagnosed with cancer, when compared to controls. Similar distributions and relationships were observed in the national sample (Online Resource 1; Table S1).

### Exposure surfaces

Figure 1 illustrates the spatial surfaces of GEM-MACH-PAH derived fluoranthene estimates applied to all participant residential histories. This figure includes exposure surfaces for both the 2.5 km × 2.5 km resolution “Pan Am” model (above), applied for Ontario-specific analyses, as well as the 10 km × 10 km resolution model (below) applied for national analyses. The spatial bounds (borders) of the nested “Pan Am” model are not displayed in this figure, but can be found elsewhere [49].

Table 2 summarizes study participant average fluoranthene exposures for both the Ontario (2.5 km model resolution) and national (10 km model resolution) samples. In general, average fluoranthene exposures were somewhat higher among participants in the Ontario-only sample, and the range of exposures was also greater in this sample. The greater range of exposure is largely attributable to the application of the finer resolution (2.5 km × 2.5 km) exposure surface which more accurately depicts areas of high or low concentration (i.e., “hot-spots” or “cold spots”), whereas the coarser resolution (10 km × 10 km) model may act to ‘smooth’ some of these areas of high concentration in with surrounding areas of, comparatively, lower concentration (or vice versa). This process is known as a low (or high) pass filter.

**Table 2:** Distribution of average residential ambient fluoranthene exposure (µg/m^3^) across Ontario (*n* = 1,233) and national (*n* = 3,773) samples and by case–control status

Study Sample & Case–Control Status	Mean ± SD	Median (Range)	IQR	*p*^a^
Ontario (2.5 km × 2.5 km)	0.0178 ± 0.0345	0.0076 (0.7011)	0.0171	0.30
Cases (*n* = 514)	0.0185 ± 0.0401	0.0083 (0.7011)	0.0165	
Controls (*n* = 719)	0.0173 ± 0.0299	0.0069 (0.2816)	0.0175	
National (10 km × 10 km)	0.0157 ± 0.0279	0.0044 (0.2729)	0.0175	< 0.01
Cases (*n* = 1,818)	0.0162 ± 0.0280	0.0062 (0.2729)	0.0176	
Controls (*n* = 1,955)	0.0152 ± 0.0279	0.0037 (0.2304)	0.0170	

*SD* Standard deviation, *IQR* Interquartile range

^a^Bivariate *p*-value for breast cancer (Wald Chi-square test)

### Ontario analysis: Breast cancer risk and fluoranthene exposure

Table 3 presents the crude and adjusted odds ratios and corresponding 95% confidence intervals for the relationship between categories of fluoranthene exposure and breast cancer risk in the Ontario sample. We observed positive associations between long-term fluoranthene exposure and breast cancer incidence for premenopausal, but not postmenopausal women. In premenopausal women, adjusted ORs of 2.48 (95% CI: 1.29, 4.77) and 1.97 (95% CI: 0.99, 3.90) were found when comparing the two highest levels of exposure, respectively, to the lowest level of exposure. Among premenopausal women, adjusted models were indicative of elevated risks when compared to crude (age-adjusted) models.

**Table 3:** Odds ratios for the incidence of breast cancer associated with ambient fluoranthene exposure, by menopausal status, Ontario sample (*n* cases = 514,* n* controls = 719) at 2.5 km model resolution

Fluoranthene exposure level (µg/m^3^)	Cases	Controls	Crude OR^a^ (95% CI)	Adjusted OR^b^ (95% CI)
Total (Pre- and post-menopausal)	514	719		
< 0.0025	102	178	1.0 ref	1.0 ref
0.0025–0.0055	95	133	1.26 (0.87, 1.80)	1.24 (0.84, 1.82)
0.0056–0.0123	110	132	1.48 (1.04, 2.11)	1.54 (1.05, 2.26)
0.0124–0.0273	113	149	1.33 (0.94, 1.88)	1.34 (0.92, 1.96)
> 0.0273	94	127	1.36 (0.94, 1.96)	1.40 (0.94, 2.09)
Premenopausal	207	244		
< 0.0025	34	63	1.0 ref	1.0 ref
0.0025–0.0055	46	49	1.93 (1.07, 3.48)	2.15 (1.14, 4.08)
0.0056–0.0123	53	41	2.69 (1.48, 4.89)	3.43 (1.79, 6.59)
0.0124–0.0273	42	50	1.68 (0.92, 3.04)	2.48 (1.29, 4.77)
> 0.0273	32	41	1.56 (0.83, 2.94)	1.97 (0.99, 3.90)
Postmenopausal	307	475		
< 0.0025	68	115	1.0 ref	1.0 ref
0.0025–0.0055	49	84	0.98 (0.61, 1.56)	0.92 (0.54, 1.55)
0.0056–0.0123	57	91	1.08 (0.69, 1.70)	1.15 (0.70, 1.90)
0.0124–0.0273	71	99	1.22 (0.79, 1.88)	1.10 (0.67, 1.80)
> 0.0273	62	86	1.26 (0.80, 1.97)	1.24 (0.74, 2.08)

^a^Crude estimates adjusted for study design factor (age-group)

^b^Adjusted for: (Total) Age-group, years of menstruation, age at first full-term pregnancy, physical activity, body mass index, smoking pack-years, total household income, meat consumption, alcohol consumption, history of benign breast disease, immediate relative diagnosed with cancer; (Postmenopausal) Age-group, years of menstruation, age at first full-term pregnancy, age at menarche, physical activity, body mass index, smoking pack-years, alcohol consumption, history of benign breast disease, immediate relative diagnosed with cancer, meat consumption, years of education, total household income, oral contraceptive use, hormone replacement therapy; (Premenopausal) Age-group, age at menarche, body mass index, history of benign breast disease, oral contraceptive use, immediate relative diagnosed with cancer

Increasing the completeness of residential history [over the exposure window] required for study inclusion (18 years; 90% of exposure window) reduced the Ontario sample size by less than 100 participants and yielded only small changes in resulting adjusted-ORs for breast cancer risk.

**Fig. 2:**
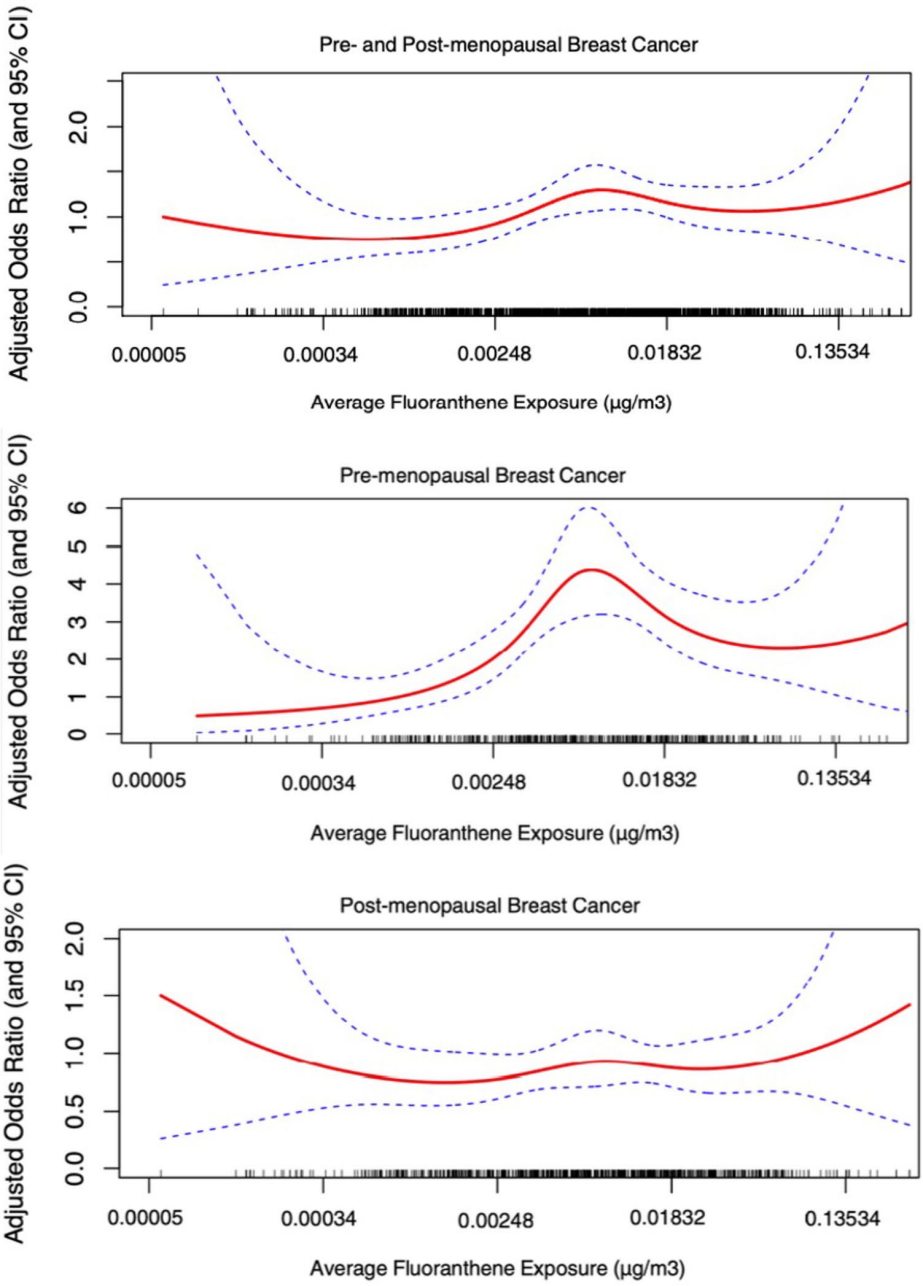
Association between the incidence of pre- and post-menopausal breast cancer and concentrations of fluoranthene using restricted cubic splines with 4 knots, Ontario sample at 2.5 km model resolution. The maximum likelihood estimate is shown as the solid line, and the broken lines represent the upper and lower pointwise 95% confidence limits. Individual spline functions adjusted for same covariates sets as for main regression analyses: (All Women) Age-group, years of menstruation, age at first full-term pregnancy, physical activity, body mass index, smoking pack-years, alcohol consumption, history of benign breast disease, immediate relative diagnosed with cancer, hormone replacement therapy; (Postmenopausal) Age-group, years of menstruation, age at first full-term pregnancy, physical activity, body mass index, smoking pack-years, alcohol consumption, history of benign breast disease, immediate relative diagnosed with cancer, meat consumption, years of education, total household income, oral contraceptive use, hormone replacement therapy; (Premenopausal) Age-group, age at menarche, body mass index, history of benign breast disease, oral contraceptive use, immediate relative diagnosed with cancer

Figure 2 displays the adjusted exposure–response splines for the Ontario sample, including relevant stratifications for menopausal status. Splines were created using restricted cubic functions with 4 degrees of freedom.

### National analysis: Breast cancer risk and fluoranthene exposure

Table 4 presents the crude and adjusted odds ratios and corresponding 95% confidence intervals for the relationship between categories of fluoranthene exposure and breast cancer risk in the national sample. We observed small, but suggestive positive associations between long-term fluoranthene exposure and breast cancer incidence for both pre- and post-menopausal women.

**Table 4:** Odds ratios for the incidence of breast cancer associated with ambient fluoranthene exposure, by menopausal status, national sample (*n* cases = 1,818,* n* controls = 1,955) at 10 km model resolution

Fluoranthene exposure level (µg/m^3^)	Cases	Controls	Crude OR^a^ (95% CI)	Adjusted OR^b^ (95% CI)
Total (Pre- and post-menopausal)	1819	1955		
< 0.0025	620	873	1.0 ref	1.0 ref
0.0025–0.0055	269	263	1.25 (1.01, 1.54)	1.23 (0.99, 1.54)
0.0056–0.0123	273	209	1.49 (1.19, 1.86)	1.41 (1.12, 1.78)
0.0124–0.0273	365	306	1.41 (1.16, 1.73)	1.36 (1.09, 1.68)
> 0.0273	292	304	1.17 (0.95, 1.45)	1.12 (0.89, 1.40)
Premenopausal	641	620		
< 0.0025	219	267	1.0 ref	1.0 ref
0.0025–0.0055	89	89	1.14 (0.79, 1.64)	1.18 (0.81, 1.71)
0.0056–0.0123	98	65	1.59 (1.08, 2.35)	1.67 (1.12, 2.49)
0.0124–0.0273	143	104	1.51 (1.07, 2.12)	1.59 (1.11, 2.29)
> 0.0273	92	95	1.04 (0.72, 1.51)	1.07 (0.72, 1.58)
Postmenopausal	1177	1335		
< 0.0025	400	606	1.0 ref	1.0 ref
0.0025–0.0055	180	174	1.30 (1.00, 1.69)	1.31 (0.99, 1.72)
0.0056–0.0123	175	144	1.45 (1.10, 1.91)	1.35 (1.02, 1.80)
0.0124–0.0273	222	202	1.37 (1.07, 1.76)	1.33 (1.02, 1.73)
> 0.0273	200	209	1.25 (0.97, 1.61)	1.27 (0.97, 1.66)

^a^Crude estimates adjusted for study design factors (age-group and study province)

^b^Adjusted for: (Total) Age-group, study province, age at menarche, years of menstruation, parity, age at first full-term pregnancy, physical activity, meat consumption, body mass index, total household income, smoking pack-years, neighborhood deprivation; (Postmenopausal) Age-group, study province, years of menstruation, parity, age at first full-term pregnancy, physical activity, meat consumption, body mass index, total household income, smoking pack-years; (Premenopausal) Age-group, study province, age at menarche, years of menstruation, parity, meat consumption, smoking pack-years, neighborhood deprivation

In premenopausal women, an adjusted OR of 1.59 (95% CI: 1.11, 2.29) was found when comparing the second-highest level of exposure to the lowest level of exposure. In postmenopausal women, a corresponding (second-highest level vs. lowest level of exposure) adjusted OR of 1.33 (95% CI: 1.02, 1.73) was found. In general, adjustment for covariates resulted in small changes in risk estimates compared to crude models.

For both national and Ontario samples, we observed a decrease in breast cancer risk associated with the highest level of exposure when compared to the second- and third-highest levels of exposure. The exception was, however, for postmenopausal women within the Ontario sample, which yielded more so a linear trend in exposure–response. These aforementioned patterns of effect were confirmed by restricted cubic spline curves for the Ontario sample (Fig. 2).

### Nitrogen dioxide

Estimates of NO_2_ exposure were available for a subset of participants based on linkage to CANUE-derived estimates of NO_2_ (year 2000) for the Ontario sample (n cases = 494, n controls = 681). The spatial correlation (Spearman) between fluoranthene and NO_2_ at individual postal codes was found to be *r*_*s*_ = 0.717. Exploratory analysis examined the risk for fluoranthene and NO_2_ in a model containing both exposures (Online Resource 1; Table S3). Effect estimates for premenopausal women were strongest for fluoranthene, and estimates for postmenopausal women were strongest for NO_2_.

## Discussion

Overall, our findings suggest an increased risk for incident breast cancer among premenopausal women exposed to higher concentrations of ambient PAHs. In comparison, findings among postmenopausal women were more mixed. Linear dose–response patterns were largely absent across both menopausal strata where positive associations were present.

The current study marks the first time that a national surface for PAH exposure has been applied to a population-based cancer study in Canada, and represents the first use of this specific exposure surface in an epidemiological context.

Despite the larger study population and domain associated with the national sample, Ontario analyses have the following two major advantages; (1) application of a much finer resolution exposure surface (i.e., less exposure misclassification, greater variability), and (2) adjustment consideration for four additional important breast cancer risk factors (i.e., oral contraceptive use, hormone replacement therapy, benign breast disease status, immediate family history of cancer). Effect estimates for premenopausal breast cancer were stronger among the Ontario sample, where the aforementioned methodological advantages were implemented.

NO_2_ is a routinely measured air pollutant and has been the subject of meta-analyses in relation to breast cancer risk [35]. However, NO_2_ is generally considered a marker for traffic-related exposures, including PAHs, rather than a causal agent. This study investigation examined fluoranthene as a more proximal marker of potential carcinogenic agents and thus it was of interest to contrast effects observed with those for NO_2_ exposure (Online Resource 1; Table S3).

Our findings, particularly concerning the contrast in association across menopausal strata, align with recently published case–control and cohort studies examining breast cancer risk with respect to residential air pollutant exposures including; Hystad et al. [44], Villeneuve et al. [38], Mordukhovich et al. [63], Goldberg et al. [64], and Nie et al. [65]. Yet, several studies have yielded positive findings for postmenopausal breast cancer [39], or have even found significant effects for postmenopausal, but not premenopausal breast cancer [37, 66]. Additionally, a few recent studies do not report any statistically significant association (for either pre- or post-menopausal breast cancer) [67–69], though these represent the minority of the published literature, especially with regard to existing work with PAHs [36, 70]. These findings relate to the fact that pre- and post-menopausal breast cancers are somewhat different diseases with varying risk factors. The pre-existing study with the most methodologically similar design, that of Amadou et al. [70], yielded an OR of 1.15 (95% CI: 1.04, 1.27) for an interquartile range (IQR) increase in benzo[*a*]pyrene exposure. Interestingly, they found that significant associations remained only for women who underwent menopausal transition (i.e., premenopausal women at recruitment who became postmenopausal at cancer diagnosis), and also noted that linear dose–response patterns were largely absent.

There are a number of methodological limitations that must be taken into account when considering our study findings. First, the retrospective (i.e., case–control) nature and provincial-based design of the current study presents some inherent potential for bias (e.g., selection bias in the recruitment of controls). With that said, and as noted by Hystad et al. who also utilized the breast cancer component of the NECSS in their study of NO_2_ [44], though case and control response rates were somewhat low, risk estimates for established risk factors obtained from the NECSS data are generally similar to what has been published in the existing literature, suggesting that the potential for selection bias in the form of participation bias is relatively low.

Second, we recognize that our exposure assignment only accounts for residential exposures, which only makes up part of total PAH exposure. With that said, the average Canadian spends a substantial proportion of their day within and in close proximity to their place of residence [71], and research with NO_2_ has shown a moderate degree of correlation between residential and total personal exposure [72]. Third, despite a comprehensive set of breast cancer risk factors considered for model adjustments, there remains the potential for residual or missing confounding. For example, our study lacked information on genetic history or predisposition for breast cancer (e.g., *BRCA1/BRCA2*), though this would likely not be related to air pollution exposure, and therefore may not be a true confounder.

Fourth, we generated PAH exposure surfaces using the best available information to represent the years of 1973–1995 (i.e., possible range of exposure years) to derive estimates of average long-term exposure. The surfaces were based on gridded emissions estimates from the earliest year (2000) combined with meteorological data from 1994 which was found to best represent average conditions over most of the exposure period [73]. In comparison to more commonly-studied constituents of air pollution (e.g., PM_2.5_, NO_2_), there is a general lack of historical fixed-site monitoring data and exposure-assignment methods for which ambient PAH exposures can be assigned. This results in challenges in generating exposure within and/or prior to the NECSS study period. While we recognize that absolute concentrations of ambient PAHs have decreased over time across the Canadian domain [74], ambient observation records rarely pre-date 2000 [75]. Despite this, limited available information is consistent with a spatial contrast in ambient concentration that has largely been maintained, as has been shown for NO_2_ [albeit, in Europe and for a shorter time period] [76]. In addition to the aforementioned limitation, there is likely further non-differential exposure misclassification associated with the use of geocoded addresses (based on postal codes) as well as the use of an ecologically-derived measure of exposure as a proxy for personal exposure. Due to this, our results likely underestimate true estimates of risk [72].

Our findings should not be interpreted as definitive causal agents with respect to breast cancer risk. PAHs may be a proxy for the complex mixture of ambient by-products derived from various combustion sources (including NO_2_) [39, 77], though the proposed biological mechanisms (i.e., endocrine-disrupting activity, DNA adduct formation), previous epidemiological studies of various cancer sites, and the presence of both particle- and gas-phase states make PAHs a particularly plausible, and proximal agent for the carcinogenesis-related effects imparted through air pollutant exposures. Additionally, it is possible that there exist certain critical periods of exposure throughout the lifetime (e.g., early-age) whereby PAH [and other ambient pollutant] exposures may be especially relevant to [breast] cancer development, which we were unable to account for in the present study [39].

In spite of limitations, this study adds to the limited existing literature of ambient PAH exposures and breast cancer risk and has a number of substantial strengths, including; (1) the first epidemiological application of a newly-developed PAH exposure surface – broadening evidence beyond criteria air pollutants, (2) detailed lifetime residential histories – reducing the potential for exposure misclassification when compared to truncated histories, (3) a high number and quality of available covariates (with specific relation to breast cancer), and (4) oversampling of premenopausal breast cancer cases – allowing for additional power when examining associations by menopausal status.

We found an association between exposure to ambient PAHs (represented by fluoranthene) and the incidence of premenopausal breast cancer among Canadian women. Associations among postmenopausal women were inconclusive given inconsistent findings across national and Ontario-specific analyses. Future research should continue to attempt to elucidate the nature of the relationship between ambient PAH exposures and breast cancer, along with other non-respiratory cancer sites, given expanding evidence for an association. Subsequent work in this area may also benefit from larger and prospective studies of breast cancer (premenopausal in particular), improved and modern exposure assessment methods for unsubstituted (‘parent’ compounds consisting of only carbon and hydrogen) and substituted (‘parent’ compounds with additional functional groups) PAHs, multi-pollutant analyses, and investigation into potential critical periods of exposure.

### Supplementary Information

Below is the link to the electronic supplementary material.

Supplementary file1 (DOCX 46 kb)

## Data Availability

The primary NECSS datasets analysed during the current study are not publicly available due to the potential for compromise of individual privacy information (including place(s) of residence). Aforementioned datasets were sourced via Dr. Paul Villeneuve’s direct research contact with Health Canada. Supplementary datasets used throughout analyses, including Canadian data on neighbourhood deprivation, particulate matter, and nitrogen dioxide can be found at https://canue.ca.

## References

[1] Sung H, Ferlay J, Siegel RL, Laversanne M, Soerjomataram I, Jemal A (2021) Global cancer statistics 2020: GLOBOCAN estimates of incidence and mortality worldwide for 36 cancers in 185 countries. CA Cancer J Clin 71:209–249. 10.3322/caac.21660 33538338 10.3322/caac.21660

[2] Lima SM, Kehm RD, Terry MB (2021) Global breast cancer incidence and mortality trends by region, age-groups, and fertility patterns. eClinicalMedicine. 10.1016/j.eclinm.2021.100985 34278281 10.1016/j.eclinm.2021.100985 PMC8271114

[3] Xu S, Liu Y, Zhang T et al (2021) The global, regional, and national burden and trends of breast cancer from 1990 to 2019: results from the global burden of disease study 2019. Front Oncol. 10.3389/fonc.2021.689562 34094989 10.3389/fonc.2021.689562 PMC8176863

[4] Global Burden of Disease (2019) Cancer Collaboration (2022) Cancer incidence, mortality, years of life lost, years lived with disability, and disability-adjusted life years for 29 cancer groups from 2010 to 2019: a systematic analysis for the global burden of disease study 2019. JAMA Oncol 8(3):420–444. 10.1001/jamaoncol.2021.6987 10.1001/jamaoncol.2021.6987 PMC8719276 34967848

[5] Canadian Cancer Society (2022) Breast cancer statistics. https://cancer.ca/en/cancer-information/cancer-types/breast/statistics. Accessed June 7, 2023

[6] Iwasaki M, Tsugane S (2011) Risk factors for breast cancer: epidemiological evidence from Japanese studies. Cancer Sci 102(9):1607–1614. 10.1111/j.1349-7006.2011.01996.x 21624009 10.1111/j.1349-7006.2011.01996.x

[7] Hulka BS, Moorman PG (2008) Breast cancer: hormones and other risk factors. Maturitas 61(1–2):203–213. 10.1016/j.maturitas.2008.11.016 19434892 10.1016/j.maturitas.2008.11.016

[8] Centers for Disease Control and Prevention (2023) What are the risk factors for breast cancer? https://www.cdc.gov/cancer/breast/basic_info/risk_factors.htm. Accessed June 7, 2023

[9] Tamimi RM, Spiegelman D, Smith-Warner SA et al (2016) Population attributable risk of modifiable and nonmodifiable breast cancer risk factors in postmenopausal breast cancer. Am J Epidemiol 184(12):884–893. 10.1093/aje/kww145 27923781 10.1093/aje/kww145 PMC5161087

[10] Heer E, Harper A, Escandor N, Sung H, McCormack V, Fidler-Benaoudia MM (2020) Global burden and trends in premenopausal and postmenopausal breast cancer: a population-based study. Lancet Glob Health 8(8):e1027–e1037. 10.1016/S2214-109X(20)30215-1 32710860 10.1016/S2214-109X(20)30215-1

[11] Canadian Cancer Society (2022) Risks for breast cancer. https://cancer.ca/en/cancer-information/cancer-types/breast/risks. Accessed June 7, 2023

[12] World Health Organization (2023) Breast cancer. https://www.who.int/news-room/fact-sheets/detail/breast-cancer. Accessed June 7, 2023

[13] Feng Y, Spezia M, Huang S et al (2018) Breast cancer development and progression: risk factors, cancer stem cells, signaling pathways, genomics, and molecular pathogenesis. Genes Dis 5(2):77–106. 10.1016/j.gendis.2018.05.001 30258937 10.1016/j.gendis.2018.05.001 PMC6147049

[14] Łukasiewicz S, Czeczelewski M, Forma A, Baj J, Sitarz R, Stanisławek A (2021) Breast cancer—epidemiology, risk factors, classification, prognostic markers, and current treatment strategies—an updated review. Cancers (Basel) 13(17):4287. 10.3390/cancers13174287 34503097 10.3390/cancers13174287 PMC8428369

[15] Engmann NJ (2019) Errors in statistical programming for study about population-attributable risk proportion of clinical risk factors for breast cancer. JAMA Oncol 5(11):1637. 10.1001/jamaoncol.2019.4355 31556914 10.1001/jamaoncol.2019.4355

[16] Coyle YM (2004) The effect of environment on breast cancer risk. Breast Cancer Res Treat 84(3):273–288. 10.1023/B:BREA.0000019964.33963.09 15026625 10.1023/B:BREA.0000019964.33963.09

[17] Rodgers KM, Udesky JO, Rudel RA, Brody JG (2018) Environmental chemicals and breast cancer: an updated review of epidemiological literature informed by biological mechanisms. Environ Res 160:152–182. 10.1016/j.envres.2017.08.045 28987728 10.1016/j.envres.2017.08.045

[18] Interagency Breast Cancer and Environmental Research Coordinating Committee (2013) Breast cancer and the environment: prioritizing prevention. https://www.niehs.nih.gov/about/assets/docs/breast_cancer_and_the_environment_prioritizing_prevention_508.pdf. Accessed June 8, 2023

[19] Boström CE, Gerde P, Hanberg A et al (2002) Cancer risk assessment, indicators, and guidelines for polycyclic aromatic hydrocarbons in the ambient air. Environ Health Perspect 110:451–488. 10.1289/ehp.110-1241197 12060843 10.1289/ehp.110-1241197 PMC1241197

[20] Kelly JM, Ivatt PD, Evans MJ et al (2021) Global cancer risk from unregulated polycyclic aromatic hydrocarbons. Geohealth. 10.1029/2021GH000401 34589640 10.1029/2021GH000401 PMC8460132

[21] Kim KH, Jahan SA, Kabir E, Brown RJ (2013) A review of airborne polycyclic aromatic hydrocarbons (PAHs) and their human health effects. Environ Int 60:71–80. 10.1016/j.envint.2013.07.019 24013021 10.1016/j.envint.2013.07.019

[22] Berthiaume A, Galarneau E, Marson G (2021) Polycyclic aromatic compounds (PACs) in the Canadian environment: sources and emissions. Environ Pollut 269:116008. 10.1016/j.envpol.2020.116008 33229050 10.1016/j.envpol.2020.116008

[23] Sofowote UM, Hung H, Rastogi AK et al (2011) Assessing the long-range transport of PAH to a sub-arctic site using positive matrix factorization and potential source contribution function. Atmos Environ 45(4):967–976. 10.1016/j.atmosenv.2010.11.005 10.1016/j.atmosenv.2010.11.005

[24] Wang X, Studens K, Parisien M-A et al (2020) Projected changes in fire size from daily spread potential in Canada over the 21st Century. Environ Res Lett 15(10):104048. 10.1088/1748-9326/aba101 10.1088/1748-9326/aba101

[25] Santonicola S, De Felice A, Cobellis L et al (2017) Comparative study on the occurrence of polycyclic aromatic hydrocarbons in breast milk and infant formula and risk assessment. Chemosphere 175:383–390. 10.1016/j.chemosphere.2017.02.084 28236708 10.1016/j.chemosphere.2017.02.084

[26] Henkler F, Stolpmann K, Luch A (2012) Exposure to polycyclic aromatic hydrocarbons: bulky DNA adducts and cellular responses. Exp Suppl 101:107–131. 10.1007/978-3-7643-8340-4_5 22945568 10.1007/978-3-7643-8340-4_5

[27] Agudo A, Peluso M, Munnia A et al (2017) Aromatic DNA adducts and breast cancer risk: a case-cohort study within the EPIC-Spain. Carcinogenesis 38(7):691–698. 10.1093/carcin/bgx047 28535209 10.1093/carcin/bgx047

[28] Santodonato J (1997) Review of the estrogenic and antiestrogenic activity of polycyclic aromatic hydrocarbons: relationship to carcinogenicity. Chemosphere 34(4):835–848. 10.1016/s0045-6535(97)00012-x 9569946 10.1016/s0045-6535(97)00012-x

[29] Wenger D, Gerecke AC, Heeb NV et al (2009) In vitro estrogenicity of ambient particulate matter: contribution of hydroxylated polycyclic aromatic hydrocarbons. J Appl Toxicol 29(3):223–232. 10.1002/jat.1400 19021152 10.1002/jat.1400

[30] Kummer V, Masková J, Zralý Z et al (2008) Estrogenic activity of environmental polycyclic aromatic hydrocarbons in uterus of immature Wistar rats. Toxicol Lett 180(3):212–221. 10.1016/j.toxlet.2008.06.862 18634860 10.1016/j.toxlet.2008.06.862

[31] Cohen AJ (2000) Outdoor air pollution and lung cancer. Environ Health Perspect 108:743–750. 10.1289/ehp.00108s4743 10931793 10.1289/ehp.00108s4743 PMC1637685

[32] Raaschou-Nielsen O, Andersen ZJ, Beelen R et al (2013) Air pollution and lung cancer incidence in 17 European cohorts: prospective analyses from the European Study of Cohorts for Air Pollution Effects (ESCAPE). Lancet Oncol 14(9):813–822. 10.1016/S1470-2045(13)70279-1 23849838 10.1016/S1470-2045(13)70279-1

[33] Loomis D, Grosse Y, Lauby-Secretan B et al (2013) The carcinogenicity of outdoor air pollution. Lancet Oncol 14(13):1262–1263. 10.1016/s1470-2045(13)70487-x 25035875 10.1016/s1470-2045(13)70487-x

[34] Turner MC, Andersen ZJ, Baccarelli A et al (2020) Outdoor air pollution and cancer: an overview of the current evidence and public health recommendations. CA Cancer J Clin. 10.3322/caac.21632 32964460 10.3322/caac.21632 PMC7904962

[35] Praud D, Deygas F, Amadou A et al (2023) Traffic-related air pollution and breast cancer risk: a systematic review and meta-analysis of observational studies. Cancers (Basel) 15(3):927. 10.3390/cancers15030927 36765887 10.3390/cancers15030927 PMC9913524

[36] Gamboa-Loira B, López-Carrillo L, Mar-Sánchez Y, Stern D, Cebrián ME (2022) Epidemiologic evidence of exposure to polycyclic aromatic hydrocarbons and breast cancer: a systematic review and meta-analysis. Chemosphere 290:133237. 10.1016/j.chemosphere.2021.133237 34929281 10.1016/j.chemosphere.2021.133237

[37] Terre-Torras I, Recalde M, Díaz Y et al (2022) Air pollution and green spaces in relation to breast cancer risk among pre and postmenopausal women: a mega cohort from Catalonia. Environ Res 214(Pt 1):113838. 10.1016/j.envres.2022.113838 35810806 10.1016/j.envres.2022.113838

[38] Villeneuve PJ, Goldberg MS, Crouse DL et al (2018) Residential exposure to fine particulate matter air pollution and incident breast cancer in a cohort of Canadian women. Environ Epi. 10.1097/ee9.0000000000000021 10.1097/ee9.0000000000000021

[39] Crouse DL, Goldberg MS, Ross NA, Chen H, Labrèche F (2010) Postmenopausal breast cancer is associated with exposure to traffic-related air pollution in Montreal, Canada: a case-control study. Environ Health Perspect 118(11):1578–1583. 10.1289/ehp.1002221 20923746 10.1289/ehp.1002221 PMC2974696

[40] Johnson KC, Mao Y, Argo J, Dubois S, Semenciw R, Lava J (1998) The National Enhanced Cancer Surveillance System: a case-control approach to environment-related cancer surveillance in Canada. Environmetrics 9(5):495–504 10.1002/(SICI)1099-095X(199809/10)9:5<495::AID-ENV318>3.0.CO;2-H

[41] Mao Y, Hu J, Ugnat AM, Semenciw R, Fincham S; Canadian Cancer Registries Epidemiology Research Group (2001) Socioeconomic status and lung cancer risk in Canada. Int J Epidemiol 30(4):809–817. 10.1093/ije/30.4.809 11511609 10.1093/ije/30.4.809

[42] World Health Organization (1979) International Classification of Diseases, 9th revision. Switzerland, Geneva

[43] Villeneuve PJ, Johnson KC, Kreiger N, Mao Y (1999) Risk factors for prostate cancer: results from the Canadian National Enhanced Cancer Surveillance System The Canadian Cancer Registries Epidemiology Research Group. Cancer Causes Control 10(5):355–367. 10.1023/a:1008958103865 10530605 10.1023/a:1008958103865

[44] Hystad P, Villeneuve PJ, Goldberg MS, Crouse DL, Johnson K; Canadian Cancer Registries Epidemiology Research Group (2015) Exposure to traffic-related air pollution and the risk of developing breast cancer among women in eight Canadian provinces: a case-control study. Environ Int 74:240–248. 10.1016/j.envint.2014.09.004 25454241 10.1016/j.envint.2014.09.004

[45] Block G, Hartman AM, Naughton D (1990) A reduced dietary questionnaire: development and validation. Epidemiology 1(1):58–64. 10.1097/00001648-199001000-00013 2081241 10.1097/00001648-199001000-00013

[46] Willett WC (1998) Nutritional Epidemiology, 2nd edn. Oxford University Press

[47] Matheson FI, Dunn JR, Smith KL, Moineddin R, Glazier RH (2012) Development of the Canadian Marginalization Index: a new tool for the study of inequality. Can J Public Health 103(8 Suppl 2):S12–S16. 10.1007/BF03403823 23618065 10.1007/BF03403823 PMC6973681

[48] CanMap Postal Code Suite v2016.3. [Computer file] Markham: DMTI Spatial Inc., 2016.

[49] Crouse DL, Ross NA, Goldberg MS (2009) Double burden of deprivation and high concentrations of ambient air pollution at the neighbourhood scale in Montreal. Canada Soc Sci Med 69(6):971–981. 10.1016/j.socscimed.2009.07.010 19656603 10.1016/j.socscimed.2009.07.010

[50] Lundqvist A, Andersson E, Ahlberg I, Nilbert M, Gerdtham U (2016) Socioeconomic inequalities in breast cancer incidence and mortality in Europe-a systematic review and meta-analysis. Eur J Public Health 26(5):804–813. 10.1093/eurpub/ckw070 27221607 10.1093/eurpub/ckw070 PMC5054273

[51] CanMap Postal Code Suite. 1996. [Computer file] Markham: DMTI Spatial Inc., 1996.

[52] Institute of Medicine (2013) Breast Cancer and the Environment: A Life Course Approach. The National Academies Press.

[53] Whaley CH, Galarneau E, Makar PA et al (2018) Gem-Mach-Pah (REV2488): A new high-resolution chemical transport model for North American polycyclic aromatic hydrocarbons and benzene. Geosci Model Dev 11(7):2609–2632. 10.5194/gmd-11-2609-2018 10.5194/gmd-11-2609-2018

[54] Whaley CH, Galarneau E, Makar PA, Moran MD, Zhang J (2020) How much does traffic contribute to benzene and polycyclic aromatic hydrocarbon air pollution? Results from a high-resolution North American air quality model centred on Toronto. Canada Atmos Chem Phys 20(5):2911–2925. 10.5194/acp-20-2911-2020 10.5194/acp-20-2911-2020

[55] Karl Wuensch (2021) Binary Logistic Regression with SPSS. https://core.ecu.edu/wuenschk/MV/Multreg/Logistic-SPSS.PDF. Accessed June 19, 2023

[56] Zhang Z (2016) Missing data imputation: focusing on single imputation. Ann Transl Med 4(1):9. 10.3978/j.issn.2305-5839.2015.12.38 26855945 10.3978/j.issn.2305-5839.2015.12.38 PMC4716933

[57] Christopher Yim (2015) Imputing missing data using SAS. https://support.sas.com/resources/papers/proceedings15/3295-2015.pdf. Accessed July 6, 2023

[58] CANUE. Data portal. https://www.canuedata.ca/index.php. Accessed June 12, 2023.

[59] Hystad P, Setton E, Cervantes A et al (2011) Creating national air pollution models for population exposure assessment in Canada. Environ Health Perspect 119(8):1123–1129. 10.1289/ehp.1002976 21454147 10.1289/ehp.1002976 PMC3237350

[60] CanMap Postal Code Suite v2015.3. [computer file] Markham: DMTI Spatial Inc., 2015.

[61] Weichenthal S, Pinault LL, Burnett RT (2017) Impact of oxidant gases on the relationship between outdoor fine particulate air pollution and nonaccidental, cardiovascular, and respiratory mortality. Sci Rep. 10.1038/s41598-017-16770-y 29180643 10.1038/s41598-017-16770-y PMC5703979

[62] Hystad P, Carpiano RM, Demers PA, Johnson KC, Brauer M (2013) Neighbourhood socioeconomic status and individual lung cancer risk: evaluating long-term exposure measures and mediating mechanisms. Soc Sci Med 97:95–103. 10.1016/j.socscimed.2013.08.005 24161094 10.1016/j.socscimed.2013.08.005

[63] Mordukhovich I, Beyea J, Herring AH et al (2016) Vehicular traffic-related polycyclic aromatic hydrocarbon exposure and breast cancer incidence: The Long Island Breast Cancer Study Project (LIBCSP). Environ Health Perspect 124(1):30–38. 10.1289/ehp.1307736 26008800 10.1289/ehp.1307736 PMC4710589

[64] Goldberg MS, Villeneuve PJ, Crouse D et al (2019) Associations between incident breast cancer and ambient concentrations of nitrogen dioxide from a national land use regression model in the Canadian National Breast Screening Study. Environ Int 133(Pt B):105182. 10.1016/j.envint.2019.105182 31648153 10.1016/j.envint.2019.105182

[65] Nie J, Beyea J, Bonner MR et al (2007) Exposure to traffic emissions throughout life and risk of breast cancer: the Western New York Exposures and Breast Cancer (WEB) study. Cancer Causes Control 18(9):947–955. 10.1007/s10552-007-9036-2 17632764 10.1007/s10552-007-9036-2

[66] Amadou A, Praud D, Coudon T et al (2023) Long-term exposure to nitrogen dioxide air pollution and breast cancer risk: A nested case-control within the French E3N cohort study. Environ Pollut 317:120719. 10.1016/j.envpol.2022.120719 36435283 10.1016/j.envpol.2022.120719

[67] Reding KW, Young MT, Szpiro AA et al (2015) Breast cancer risk in relation to ambient air pollution exposure at residences in the Sister Study cohort. Cancer Epidemiol Biomarkers Prev 24(12):1907–1909. 10.1158/1055-9965.EPI-15-0787 26464427 10.1158/1055-9965.EPI-15-0787 PMC4686338

[68] Andersen ZJ, Ravnskjær L, Andersen KK et al (2017) Long-term exposure to fine particulate matter and breast cancer incidence in the Danish Nurse cohort study. Cancer Epidemiol Biomarkers Prev 26(3):428–430. 10.1158/1055-9965.EPI-16-0578 27913396 10.1158/1055-9965.EPI-16-0578

[69] Hart JE, Bertrand KA, DuPre N et al (2016) Long-term particulate matter exposures during adulthood and risk of breast cancer incidence in the Nurses’ Health Study II prospective cohort. Cancer Epidemiol Biomarkers Prev 25(8):1274–1276. 10.1158/1055-9965.EPI-16-0246 27257091 10.1158/1055-9965.EPI-16-0246 PMC4970922

[70] Amadou A, Praud D, Coudon T et al (2021) Risk of breast cancer associated with long-term exposure to benzo[a]pyrene (BaP) air pollution: Evidence from the French E3N cohort study. Environ Int 149:106399. 10.1016/j.envint.2021.106399 33503556 10.1016/j.envint.2021.106399

[71] Canadian Fitness and Lifestyle Research Institute (1996) How Canadians spend their time. Progress in Prevention, Bulletin no. 4. https://cflri.ca/sites/default/files/node/151/files/pip06.pdf. Accessed January 5, 2024.

[72] Stroh E, Rittner R, Oudin A et al (2012) Measured and modeled personal and environmental NO2 exposure. Popul Health Metr 10(1):10. 10.1186/1478-7954-10-10 22681784 10.1186/1478-7954-10-10 PMC3463478

[73] Pentcheva N (2022) 1980–1999 Meteorological analysis. Report prepared by the Meteorological Service of Canada, Environment and Climate Change Canada, Montreal, QC.

[74] Li W, Park R, Alexandrou N, Dryfhout-Clark H, Brice K, Hung H (2021) Multi-year analyses reveal different trends, sources, and implications for source-related human health risks of atmospheric polycyclic aromatic hydrocarbons in the Canadian Great Lakes Basin. Enviorn Sci Technol 55(4):2254–2264. 10.1021/acs.est.0c07079 10.1021/acs.est.0c07079 33512990

[75] Tevlin A, Galarneau E, Zhang T, Hung H (2021) Polycyclic aromatic compounds (PACs) in the Canadian environment: ambient air and deposition. Environ Pollut 271:116232. 10.1016/j.envpol.2020.116232 33412446 10.1016/j.envpol.2020.116232

[76] Eeftens M, Beelen R, Fischer P, Brunekreef B, Meliefste K, Hoek G (2011) Stability of measured and modelled spatial contrasts in NO2 over time. Occup Environ Med 68(10):765–770. 10.1136/oem.2010.061135 21285243 10.1136/oem.2010.061135

[77] Goldberg MS (2007) On the interpretation of epidemiological studies of ambient air pollution. J Expo Sci Environ Epidemiol. 10.1038/sj.jes.7500629 18079766 10.1038/sj.jes.7500629

