# Supplementary material for: Ambient polycyclic aromatic hydrocarbon exposure and breast cancer risk in a population-based Canadian case–control study
Source: Cancer Causes Control. 2024 Apr 17;35(8):1165–80. doi: 10.1007/s10552-024-01866-4 (PMC11266283; doi:10.1007/s10552-024-01866-4)
Supplement: Supplementary file 1 — Supplementary file1 (DOCX 46 kb) [file 10552_2024_1866_MOESM1_ESM.docx]

**Supplemental Information**

**Ambient Polycyclic Aromatic Hydrocarbon Exposure and Breast Cancer Risk in a Population-Based Canadian Case-Control Study**

Journal: *Cancer Causes & Control*

Patrick Hinton,^1^ Paul J. Villeneuve,^2^ Elisabeth Galarneau,^3^ Kristian Larsen,^4^ Deyong Wen,^3^ Jun Meng,^3^ Verica Savic-Jovcic,^3^ Junhua Zhang,^3^ Will D King^1^

1. Department of Public Health Sciences, Queen's University, Kingston, Ontario, Canada.
2. Department of Neuroscience, Carleton University, Ottawa, Ontario, Canada.
3. Air Quality Research Division, Environment and Climate Change Canada, Toronto, Ontario, Canada.
4. Office of Environmental Health, Health Canada, Ottawa, Ontario Canada.

Corresponding Author: Will D King ([kingw@queensu.ca](mailto:kingw@queensu.ca))

**Table S1** Distribution of breast cancer risk factors by case-control and menopausal status, national sample, (*n* cases = 1818, *n* controls = 1955)

| Variable*^a^* | Pre-menopausal*^b^* |  |  | Post-menopausal*^b^* |  |  |
| --- | --- | --- | --- | --- | --- | --- |
|  | Cases  [*n* (%)] | Controls  [*n* (%)] | *P* *^c^* | Cases  [*n* (%)] | Controls  [*n* (%)] | *P* *^c^* |
| Age group, years |  |  | <0.01 |  |  | 0.46 |
| 20-29 | 6 (0.9) | 39 (6.3) |  | 0 (0) | 1 (0.1) |  |
| 30-39 | 99 (15.4) | 99 (16.0) |  | 8 (0.7) | 2 (0.2) |  |
| 40-49 | 360 (56.2) | 301 (48.6) |  | 84 (7.1) | 84 (6.3) |  |
| 50-59 (ref.) | 151 (23.6) | 136 (21.9) |  | 305 (25.9) | 328 (24.6) |  |
| 60-69 | 24 (3.7) | 43 (6.9) |  | 502 (42.7) | 602 (45.1) |  |
| >70 | 0 (0) | 0 (0) |  | 277 (23.5) | 318 (23.8) |  |
| Not Reported | 1 (0.2) | 2 (0.3) |  | 1 (0.1) | 0 (0) |  |
| Study Province |  |  | <0.01 |  |  | <0.01 |
| British Columbia (BC) | 186 (29.0) | 99 (16.0) |  | 432 (36.7) | 215 (16.1) |  |
| Alberta (AB) | 70 (10.9) | 69 (11.1) |  | 127 (10.8) | 157 (11.8) |  |
| Saskatchewan (SK) | 27 (4.2) | 26 (4.2) |  | 76 (6.5) | 74 (5.5) |  |
| Manitoba (MN) | 33 (5.2) | 37 (6.0) |  | 67 (5.7) | 93 (7.0) |  |
| Ontario (ON) (ref.) | 224 (35.0) | 260 (41.9) |  | 312 (26.5) | 489 (36.6) |  |
| Nova Scotia (NS) | 27 (4.2) | 49 (7.9) |  | 65 (5.5) | 155 (11.6) |  |
| Prince Edward Island (PE) | 42 (6.6) | 46 (7.4) |  | 71 (6.0) | 75 (5.6) |  |
| Newfoundland and Labrador (NF) | 32 (5.0) | 34 (5.5) |  | 27 (2.3) | 77 (5.8) |  |
| Body mass index (kg/m^2^) |  |  | 0.73 |  |  | 0.01 |
| <18.5 | 20 (3.1) | 21 (3.4) |  | 23 (2.0) | 27 (2.0) |  |
| 18.5 to <25 (ref.) | 379 (59.1) | 368 (59.4) |  | 550 (46.7) | 684 (51.2) |  |
| 25 to <30 | 151 (23.6) | 153 (24.7) |  | 377 (32.0) | 433 (32.4) |  |
| ≥30 | 87 (13.6) | 77 (12.4) |  | 225 (19.1) | 185 (13.9) |  |
| Not Reported | 4 (0.6) | 1 (0.2) |  | 2 (0.2) | 6 (0.5) |  |
| Years of education |  |  | 0.29 |  |  | 0.01 |
| <11 (ref.) | 83 (13.0) | 99 (15.6) |  | 385 (32.7) | 502 (37.6) |  |
| 11-12 | 228 (35.6) | 188 (30.3) |  | 374 (31.8) | 421 (31.5) |  |
| 13-14 | 127 (19.8) | 132 (21.3) |  | 187 (15.9) | 208 (15.6) |  |
| >14 | 201 (31.4) | 199 (32.1) |  | 217 (18.4) | 185 (13.9) |  |
| Not Reported | 2 (0.3) | 2 (0.3) |  | 14 (1.2) | 19 (1.4) |  |
| Total household income (CAD$) |  |  | 0.08 |  |  | 0.01 |
| <$50,000 (ref.) | 252 (39.3) | 259 (41.8) |  | 623 (52.9) | 741 (55.5) |  |
| $50,000-$99,999 | 223 (34.8) | 182 (29.4) |  | 192 (16.3) | 159 (11.9) |  |
| ≥$100,000 | 48 (7.5) | 39 (6.3) |  | 22 (1.9) | 40 (3.0) |  |
| Not Reported | 118 (18.4) | 140 (22.6) |  | 340 (28.9) | 395 (29.6) |  |
| Physical activity (standardize MET score quartiles)*^d^* |  |  | 0.31 |  |  | 0.10 |
| Q1 (< -0.73) (ref.) | 141 (22.0) | 138 (22.3) |  | 291 (24.7) | 320 (24.0) |  |
| Q2 (-0.73 to -0.12) | 172 (26.8) | 154 (24.8) |  | 277 (23.5) | 280 (21.0) |  |
| Q3 (-0.13 to 0.54) | 148 (23.1) | 126 (20.3) |  | 282 (24.0) | 322 (24.1) |  |
| Q4 (> 0.54) | 146 (22.8) | 155 (25.0) |  | 249 (21.2) | 338 (25.3) |  |
| Not Reported | 34 (5.3) | 47 (7.6) |  | 78 (6.6) | 75 (5.6) |  |
| Index of neighbourhood deprivation (quintiles)*^e^* |  |  | 0.02 |  |  | <0.01 |
| Q1 (Most deprived) (ref.) | 147 (22.9) | 101 (16.3) |  | 256 (21.8) | 192 (14.4) |  |
| Q2 | 130 (20.3) | 137 (22.1) |  | 230 (19.5) | 278 (20.8) |  |
| Q3 | 140 (21.8) | 134 (21.6) |  | 274 (23.3) | 295 (22.1) |  |
| Q4 | 136 (21.2) | 134 (21.6) |  | 240 (20.4) | 309 (23.2) |  |
| Q5 (Least deprived) | 88 (13.7) | 114 (18.4) |  | 177 (15.0) | 261 (19.6) |  |
| Smoking pack-years |  |  | 0.14 |  |  | <0.01 |
| 0 (ref.) | 296 (46.2) | 310 (50.0) |  | 549 (46.6) | 690 (51.7) |  |
| 1 | 64 (10.0) | 42 (6.8) |  | 49 (4.2) | 59 (4.4) |  |
| 2-13 | 167 (26.1) | 146 (23.6) |  | 196 (16.7) | 243 (18.2) |  |
| >13 | 103 (16.1) | 114 (18.4) |  | 365 (31.0) | 321 (24.0) |  |
| Not Reported | 11 (1.7) | 8 (1.3) |  | 18 (1.5) | 22 (1.7) |  |
| Alcohol consumption (units/week) |  |  | 0.16 |  |  | 0.09 |
| 0 (ref.) | 235 (36.7) | 264 (42.6) |  | 552 (46.9) | 655 (49.1) |  |
| <0.5 | 70 (10.9) | 69 (11.1) |  | 115 (9.8) | 156 (11.7) |  |
| 0.5-3.5 | 174 (27.2) | 148 (23.9) |  | 250 (21.2) | 275 (20.6) |  |
| >3.5 | 162 (25.3) | 139 (22.4) |  | 260 (22.1) | 249 (18.7) |  |
| Meat consumption (servings/week; quartiles) |  |  | 0.29 |  |  | 0.01 |
| Q1 (<3.87) (ref.) | 120 (18.7) | 133 (21.5) |  | 301 (25.6) | 382 (28.6) |  |
| Q2 (3.87-6.40) | 141 (22.0) | 153 (24.7) |  | 297 (25.2) | 338 (25.3) |  |
| Q3 (6.41-9.81) | 189 (29.5) | 167 (26.9) |  | 266 (22.6) | 337 (25.2) |  |
| Q4 (>9.81) | 191 (29.8) | 167 (26.9) |  | 313 (26.6) | 278 (20.8) |  |
| Vegetable consumption (servings/week; quartiles) |  |  | 0.55 |  |  | 0.57 |
| Q1 (<13.90) (ref.) | 172 (26.8) | 183 (29.5) |  | 246 (20.9) | 309 (23.2) |  |
| Q2 (13.90-18.96) | 185 (28.9) | 173 (27.9) |  | 274 (23.3) | 311 (23.3) |  |
| Q3 (18.97-25.46) | 147 (22.9) | 148 (23.9) |  | 308 (26.2) | 337 (25.2) |  |
| Q4 (>25.46) | 137 (21.4) | 116 (18.7) |  | 349 (29.7) | 378 (28.3) |  |
| Parity |  |  | 0.21 |  |  | <0.01 |
| 0 (ref.) | 91 (14.2) | 104 (16.8) |  | 153 (13.0) | 112 (8.4) |  |
| 1 | 68 (10.6) | 51 (8.2) |  | 90 (7.7) | 87 (6.5) |  |
| 2 | 203 (31.7) | 189 (30.5) |  | 230 (19.5) | 269 (20.2) |  |
| 3 | 154 (24.0) | 134 (21.6) |  | 279 (23.7) | 255 (19.1) |  |
| ≥4 | 125 (19.5) | 142 (22.9) |  | 424 (36.0) | 609 (45.6) |  |
| Not Reported | 0 (0) | 0 (0) |  | 1 (0.1) | 3 (0.2) |  |
| Ever breast fed |  |  | 0.52 |  |  | <0.01 |
| No (ref.) | 197 (30.7) | 201 (32.4) |  | 376 (32.0) | 513 (38.4) |  |
| Yes | 444 (69.3) | 419 (67.6) |  | 801 (68.1) | 821 (61.5) |  |
| Not Reported | 0 (0) | 0 (0) |  | 0 (0) | 1 (0.1) |  |
| Age at first full-term pregnancy, years |  |  | 0.10 |  |  | <0.01 |
| <18 | 27 (4.2) | 23 (3.7) |  | 42 (3.6) | 50 (3.8) |  |
| 18-26 (ref.) | 297 (46.3) | 308 (49.7) |  | 634 (53.9) | 855 (64.0) |  |
| 27-30 | 159 (24.8) | 134 (21.6) |  | 233 (19.8) | 242 (18.1) |  |
| >30 | 66 (10.3) | 45 (7.3) |  | 108 (9.2) | 70 (5.2) |  |
| Never Pregnant | 92 (14.4) | 110 (17.7) |  | 160 (13.6) | 118 (8.8) |  |
| Years of menstruation (quartiles) |  |  | 0.03 |  |  | <0.01 |
| Q1 (<28) (ref.) | 115 (17.9) | 148 (23.9) |  | 214 (18.2) | 278 (20.8) |  |
| Q2 (28-33) | 181 (28.2) | 159 (25.7) |  | 264 (22.4) | 302 (22.6) |  |
| Q3 (34-37) | 159 (24.8) | 121 (19.5) |  | 256 (21.8) | 282 (21.1) |  |
| Q4 (>37) | 120 (18.7) | 118 (19.0) |  | 381 (32.4) | 361 (27.0) |  |
| Not Reported | 66 (10.3) | 74 (11.9) |  | 62 (5.3) | 112 (8.4) |  |
| Age at menarche, years |  |  | 0.05 |  |  | 0.01 |
| <12 (ref.) | 131 (20.4) | 102 (16.5) |  | 201 (17.1) | 211 (15.8) |  |
| 12 | 172 (26.8) | 163 (26.3) |  | 270 (22.9) | 282 (21.1) |  |
| 13 | 181 (28.2) | 156 (25.2) |  | 320 (27.2) | 323 (24.2) |  |
| 14 | 76 (11.9) | 86 (13.9) |  | 182 (15.5) | 213 (16.0) |  |
| >14 | 45 (7.0) | 66 (10.7) |  | 153 (13.0) | 213 (16.0) |  |
| Not Reported | 36 (5.6) | 47 (7.6) |  | 51 (4.3) | 93 (7.0) |  |

NE, not estimated

MET, Metabolic Equivalent of Task

**^a^**Categories shown are those modeled in regression analyses, excluding ‘not reported’ categories.

**^b^**Premenopausal women defined as women who were (at time of interview); (i) still menstruating, or (ii) menstruating status not reported and age less than 50, or (iii) not currently menstruating and last menstruation within previous year

**^c^**Bivariate p-value for breast cancer (Wald chi-square test)

**^d^**Physical activity measure for Ontario standardized in harmonization with national analysis. **^e^**Quintiles based on single component (deprivation) of long-term neighbourhood socioeconomic status index associated with participant longest residence [1]

**Table S2** Spatial correlation matrix of unsubstituted PAHs (and Benzene) across the Canadian (national) domain (10km model resolution) generated from GEM-MACH-PAH model simulation for year 2000. Analysis performed by Deyong Wen (ECCC; Personal communication: deyong.wen@ec.gc.ca)

|  | Fluoranthene | Anthracene | Benzo[a]pyrene | Phenanthrene | Benz[a]anthracene | Chrysene | Pyrene | Benzene |
| --- | --- | --- | --- | --- | --- | --- | --- | --- |
| Fluoranthene | 1.0000 | 0.9799 | 0.9964 | 0.8198 | 0.9921 | 0.9932 | 0.9901 | 0.1071 |
| Anthracene |  | 1.0000 | 0.9701 | 0.9124 | 0.9500 | 0.9959 | 0.9476 | 0.1154 |
| Benzo[a]pyrene |  |  | 1.0000 | 0.7942 | 0.9949 | 0.9858 | 0.9938 | 0.1412 |
| Phenanthrene |  |  |  | 1.0000 | 0.7433 | 0.8773 | 0.7374 | 0.1594 |
| Benz[a]anthracene |  |  |  |  | 1.0000 | 0.9725 | 0.9994 | 0.0946 |
| Chrysene |  |  |  |  |  | 1.0000 | 0.9705 | 0.1074 |
| Pyrene |  |  |  |  |  |  | 1.0000 | 0.0904 |
| Benzene |  |  |  |  |  |  |  | 1.0000 |

**Table S3** Odds ratios for the incidence of breast cancer associated with ambient NO_2_ and fluoranthene exposures, modelled individually and together, by menopausal status, Ontario sample (*n* cases = 494, *n* controls = 681)

| NO_2_ Exposure Quintiles (µg/m^3^) | Cases | Controls | Adjusted OR^a^ (95% CI) - Individually | Adjusted OR^b^ (95% CI) - Together |
| --- | --- | --- | --- | --- |
| Total (Pre- and post-menopausal) | 494 | 681 |  |  |
| <18.30 | 88 | 137 | 1.0 ref. | 1.0 ref. |
| 18.30 - 27.01 | 90 | 135 | 1.01 (0.67, 1.53) | 0.95 (0.59, 1.51) |
| 27.02 - 36.97 | 105 | 136 | 1.30 (0.87, 1.94) | 1.18 (0.70, 1.99) |
| 36.98 - 48.32 | 109 | 140 | 1.26 (0.84, 1.90) | 1.24 (0.70, 2.19) |
| >48.32 | 102 | 133 | 1.36 (0.90, 2.06) | 1.41 (0.76, 2.62) |
| Premenopausal | 198 | 230 |  |  |
| <18.30 | 33 | 43 | 1.0 ref. | 1.0 ref. |
| 18.30 - 27.01 | 39 | 52 | 1.14 (0.58, 2.26) | 0.77 (0.36, 1.66) |
| 27.02 - 36.97 | 45 | 41 | 1.64 (0.83, 3.23) | 0.85 (0.36, 1.99) |
| 36.98 - 48.32 | 44 | 46 | 1.72 (0.88, 3.39) | 1.01 (0.40, 2.56) |
| >48.32 | 37 | 48 | 1.50 (0.75, 3.02) | 1.01 (0.36, 2.81) |
| Postmenopausal | 296 | 451 |  |  |
| <18.30 | 55 | 94 | 1.0 ref. | 1.0 ref. |
| 18.30 - 27.01 | 51 | 83 | 1.11 (0.64, 1.93) | 1.21 (0.64, 2.29) |
| 27.02 - 36.97 | 60 | 95 | 1.17 (0.69, 1.97) | 1.36 (0.67, 2.76) |
| 36.98 - 48.32 | 65 | 94 | 1.09 (0.64, 1.85) | 1.32 (0.62, 2.84) |
| >48.32 | 65 | 85 | 1.53 (0.89, 2.63) | 1.91 (0.84, 4.35) |
| Fluoranthene Exposure Level (µg/m^3^) |  |  |  |  |
| Total (Pre- and post-menopausal) | 494 | 681 |  |  |
| <0.0025 | 91 | 151 | 1.0 ref. | 1.0 ref. |
| 0.0025-0.0055 | 95 | 127 | 1.22 (0.81, 1.83) | 1.13 (0.69, 1.85) |
| 0.0056-0.0123 | 107 | 130 | 1.48 (0.99, 2.21) | 1.29 (0.77, 2.16) |
| 0.0124-0.0273 | 108 | 149 | 1.21 (0.82, 1.80) | 0.95 (0.54, 1.69) |
| >0.0273 | 93 | 124 | 1.35 (0.89, 2.04) | 1.01 (0.54, 1.90) |
| Premenopausal | 198 | 230 |  |  |
| <0.0025 | 30 | 51 | 1.0 ref. | 1.0 ref. |
| 0.0025-0.0055 | 46 | 49 | 2.14 (1.10, 4.18) | 2.32 (1.04, 5.20) |
| 0.0056-0.0123 | 51 | 40 | 3.47 (1.73, 6.93) | 3.58 (1.53, 8.38) |
| 0.0124-0.0273 | 39 | 50 | 2.30 (1.15, 4.63) | 2.26 (0.84, 5.93) |
| >0.0273 | 32 | 40 | 2.02 (0.98, 4.17) | 1.86 (0.63, 5.49) |
| Postmenopausal | 296 | 451 |  |  |
| <0.0025 | 61 | 100 | 1.0 ref. | 1.0 ref. |
| 0.0025-0.0055 | 49 | 78 | 0.97 (0.56, 1.67) | 0.82 (0.42, 1.60) |
| 0.0056-0.0123 | 56 | 90 | 1.11 (0.66, 1.86) | 0.89 (0.44, 1.78) |
| 0.0124-0.0273 | 69 | 99 | 1.03 (0.62, 1.72) | 0.72 (0.34, 1.55) |
| >0.0273 | 61 | 84 | 1.20 (0.70, 2.04) | 0.80 (0.35, 1.80) |

**^a^**Adjusted for: (Total) Age-group, years of menstruation, age at first full-term pregnancy, physical activity, body mass index, smoking pack-years, total household income, meat consumption, alcohol consumption, history of benign breast disease, immediate relative diagnosed with cancer; (Postmenopausal) Age-group, years of menstruation, age at first full-term pregnancy, age at menarche, physical activity, body mass index, smoking pack-years, alcohol consumption, history of benign breast disease, immediate relative diagnosed with cancer, meat consumption, years of education, total household income, oral contraceptive use, hormone replacement therapy; (Premenopausal) Age-group, age at menarche, body mass index, history of benign breast disease, oral contraceptive use, immediate relative diagnosed with cancer

^b^Adjusted for same covariates as individual models (listed above) and for the other listed ambient pollutant (i.e., NO_2_ analysis adjusted for fluoranthene exposure, and vice versa)

**References**

1. Matheson FI, Dunn JR, Smith KL, Moineddin R, Glazier RH (2012) Development of the Canadian Marginalization Index: a new tool for the study of inequality. Can J Public Health 103(8 Suppl 2):S12-S16. <https://doi.org/10.1007/BF03403823>
